# Atypical femoral fracture after receiving antiresorptive drugs in breast cancer patients with bone metastasis

**DOI:** 10.1007/s12282-016-0746-8

**Published:** 2016-12-10

**Authors:** Seiya Ota, Ryo Inoue, Takashi Shiozaki, Yuji Yamamoto, Naoki Hashimoto, On Takeda, Kei Yoshikawa, Junji Ito, Yasuyuki Ishibashi

**Affiliations:** 1 Department of Orthopaedic Surgery, Hirosaki University Graduate School of Medicine, 5 Zaifu-cho, Hirosaki, 036-8562 Aomori Japan; 2 Department of Surgery, Aomori Prefectural Central Hospital, Aomori, Japan; 3 Department of Orthopaedic Surgery, Aomori Prefectural Central Hospital, Aomori, Japan

**Keywords:** Atypical femoral fracture, Bisphosphonate, Denosumab, Breast cancer, Beaking

## Abstract

**Background:**

Atypical femoral fracture (AFF) occurs with minor trauma in patients receiving antiresorptive drugs such as bisphosphonate and denosumab. We hypothesized that patients with bone metastasis who receive higher doses of antiresorptive drugs tend to experience AFF more frequently. This study aimed to investigate the prevalence rate of AFF in patients receiving antiresorptive drugs for bone metastasis of breast cancer.

**Methods:**

Based on the database from our hospital, patients with breast cancer between March and September 2014 were investigated. Thirty-two patients with bone metastasis who received higher doses of antiresorptive drugs were included for analysis and defined as the metastasis (M) group. For the control (C) group, 32 patients in the same period with breast cancer without bone metastasis who did not undergo antiresorptive drug therapy were included. We evaluated the localized periosteal thickening of the lateral cortex (beaking) and femoral neck-shaft angle in CT scout view, the periods from induction of antiresorptive drugs to the appearance of beaking, and the occurrence rate of complete fracture. The 2 groups were compared.

**Results:**

Of the 64 limbs in 32 patients of the M group, 8 limbs in 6 patients showed beaking at the subtrochanteric area (12.5%). After the occurrence of beaking, 5 limbs in 3 patients eventually had a complete fracture with minor trauma (7.8%). On the other hand, no beaking was observed in the C group.

**Conclusions:**

The frequency of AFF in patients with breast cancer receiving bisphosphonate and/or denosumab for bone metastasis was high. More attention should be paid to the occurrence of AFF in these patients than osteoporotic patients.

## Introduction

Atypical femoral fracture (AFF) occurs with minor trauma from the lesser trochanter to the supracondylar. This fracture configuration is transverse or short oblique, non-comminuted or minimally comminuted, and has focal or diffuse cortical thickening in the lateral cortex of the subtrochanteric or femoral shaft region. This localized periosteal or endosteal thickening of the lateral cortex is called “beaking” and is one of the major features of this fracture. Minor features are a generalized increase in cortical thickness of the femoral diaphysis, prodromal symptoms such as dull or aching pain in the groin or thigh, and delayed fracture healing [1].

AFFs have been reported as a potential complication of long-term bisphosphonate therapy for the treatment of osteoporosis [2]. There are many studies and case reports indicating that AFF could be more frequent in osteoporotic patients receiving antiresorptive drugs such as bisphosphonate and denosumab [3, 4]. Antiresorptive drugs reduce not only bone loss and risk of fractures in patients with osteoporosis [5], but also the frequency of skeletal-related events such as pathologic bone fracture, spinal cord compression, and hypercalcemia due to cancer with bone metastasis [6–8]. The doses of bisphosphonates used for bone metastasis are up to 10 times greater than those for osteoporosis [9]. Similarly, the doses of denosumab used for metastasis are greater than those for osteoporosis. If antiresorptive drugs cause AFF, patients with bone metastasis who receive a much higher dose of those drugs than patients with osteoporosis might be at higher risk for AFF. However, there have been few reports that investigated the association between AFF and antiresorptive drugs in patients with bone metastasis.

Recently, we experienced several cases of AFF caused by minor trauma in patients with breast cancer undergoing antiresorptive drug treatment. In our hospital, computed tomography (CT) is performed in breast cancer patients to follow up for distant metastasis at the department of breast surgery. CT allows sequential image evaluation and early diagnosis of AFF in patients with breast cancer. We hypothesized that patients with bone metastasis who receive higher doses of antiresorptive drugs would tend to incur AFF at earlier phases and higher rates compared to those who do not receive antiresorptive drugs. The purpose of this study was to investigate the prevalence rate of AFF in patients receiving antiresorptive drugs for bone metastasis of breast cancer.

## Materials and methods

### Patients

Based on the database from the department of breast surgery in our hospital, patients with breast cancer between March and September 2014 were investigated. Of these, 53 female patients who were treated as bone metastasis were identified. An average age of these patients was 61.3 years (range 38–86 years). Detailed review of all medical records and imaging studies was performed retrospectively, and the height, weight, body mass index (BMI), past history, hormonal therapy, serum calcium and follow-up periods after induction of antiresorptive therapy from 2006 to 2015 were identified. The inclusion criteria were as follows: (1) use of intravenous pamidronic acid and/or zoledronic acid and/or subcutaneous denosumab as treatment for bone metastasis, and (2) clinical follow-up for at least 2 years after induction of high-dose antiresorptive drugs, and (3) CT scan taken from the neck level to proximal thigh level every 6 months for at least 2 years. Patients who had a history of osteoporosis or incomplete medical records were excluded. The remaining 32 patients were included for analysis. These patients were defined as the Metastasis (M) group. For the control group, 32 patients who had breast cancer without bone metastasis, and did not undergo antiresorptive therapy in the same period were extracted. These patients were matched to the M group by sex, BMI, follow-up period and hormonal therapy, and defined as the control (C) group. The institutional review board of the Hirosaki University Graduate School of Medicine approved the protocol, and all patients and their families were informed that the data concerning their case would be submitted for publication. We obtained their consent.

### Imaging

**Fig. 1:**
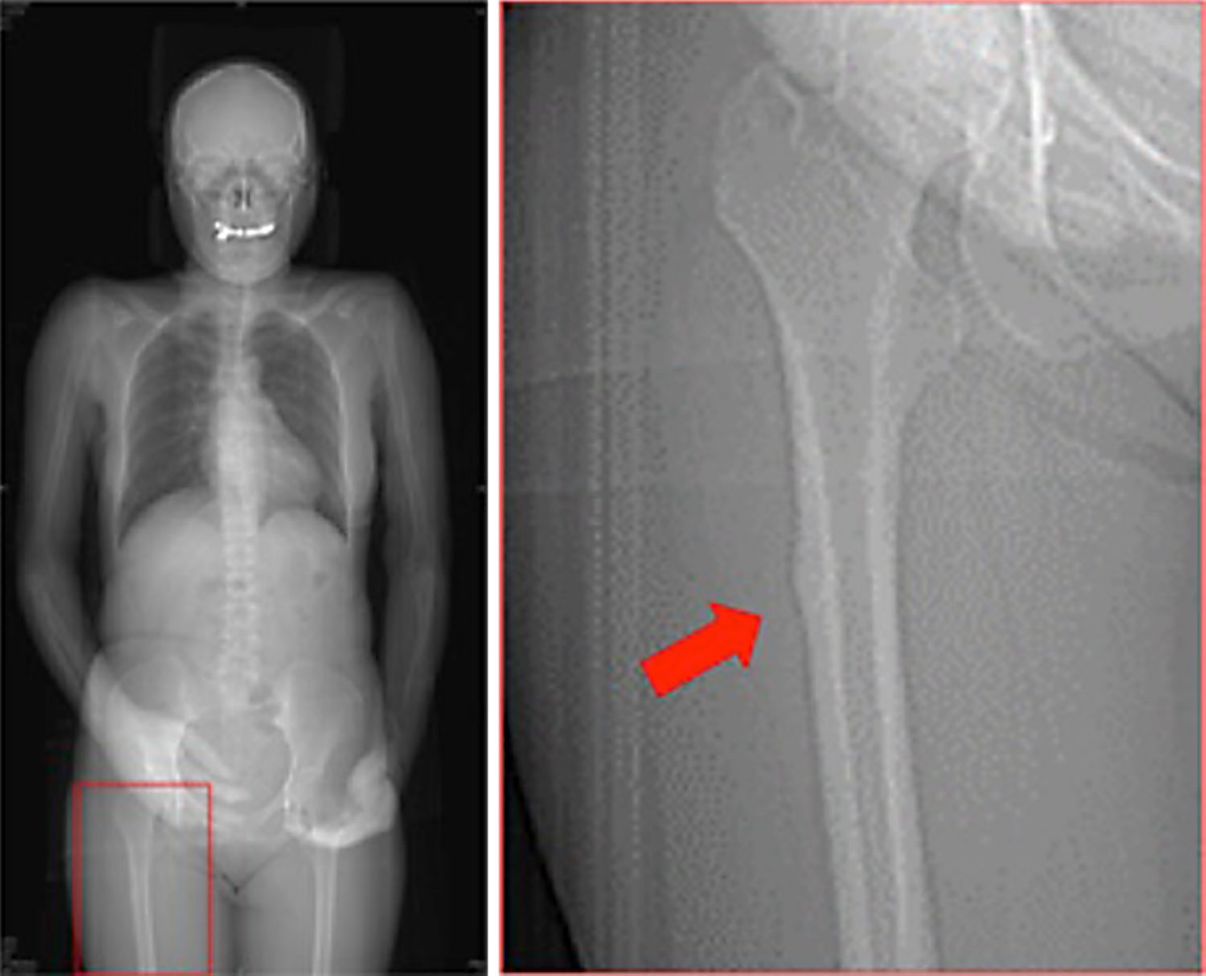
Beaking of lateral cortex in scout view of CT. The presence of localized periosteal or endosteal thickening of the lateral cortex at the subtrochanteric region (beaking) in CT scout view

To determine the presence of AFF, all patients’ CT images during the follow-up periods were evaluated by one investigator (S.O.) who knew the incidence of fractures. The checkpoint was the presence of localized periosteal or endosteal thickening of the lateral cortex at the subtrochanteric region (beaking) in CT scout view (Fig. 1). Beaking was defined as the presence of cortex extrusion. When it was difficult to judge beaking on the CT scout view, a senior orthopedic surgeon (R.I.) was consulted and a consensus was reached. After detection of beaking on CT, the periods from induction of antiresorptive agents to the appearance of beaking, the occurrence rate of complete fracture, and the period from appearance of beaking to the occurrence of complete fracture were investigated. The femoral neck-shaft angle was measured in CT scout view. The femoral neck-shaft angle was defined as the angle formed by the intersection of a line down the centre of the femoral neck and a line through the centre of the femoral shaft. Bony union was defined as bridging of 3 of the 4 cortices on AP and lateral radiographs [10].

### Statistical analysis

Data input and calculation were performed with the SPSS ver.12.0 J (SPSS Inc., Chicago, IL, USA). The comparison of age, height, body weight, BMI, follow-up period, and neck-shaft angle between the M group and C group was performed using the Student’s *t* test for parametric data and Mann–Whitney *U* test for nonparametric data. Correlations of treatment duration of antiresorptive drugs and beaking or AFF were investigated with Spearman’s correlation. In all analyses, *P* values <0.05 were considered significant.

## Results

Characteristics of the M and C groups are shown in Table 1. The average age of the M group was 59.2 ± 11.6 years, height was 152.8 ± 6.7 cm, weight was 54.2 ± 8.8 kg, BMI was 23.2 ± 3.6 kg/m^2^, and follow-up period was 56.6 ± 25.7 months. On the other hand, the average age of the C group was 61.9 ± 13.4 years, height was 154.4 ± 5.8 cm, weight was 56.5 ± 9.4 kg, BMI was 23.7 ± 3.6 kg/m^2^, and follow-up period was 53.5 ± 27.4 months. There were no significant differences in age (*p* = 0.444), height (*p* = 0.485), body weight (*p* = 0.452), BMI (*p* = 0.677), and follow-up period (*p* = 0.528) between the 2 groups. One patient of the M group underwent pamidronic acid and zoledronic acid therapy, 12 underwent zoledronic acid only, 18 underwent zoledronic acid and denosumab, and 1 underwent denosumab only (Table 2). These antiresorptive drugs were not used concomitantly. Patients typically received 4 mg zoledronic acid or 120 mg denosumab every 3–4 weeks. The average of administration period of zoledronic acid was 43.5 ± 28.0 months. That of denosumab was 27.6 ± 9.7 months. There were many variations of past history in both groups, such as hypertension, diabetes, hyperlipidaemia, angina, cerebral haemorrhage and infarction, tuberculosis, cholecystitis, asthma, systemic lupus erythematosus, rheumatoid arthritis, Hashimoto’s thyroiditis, Sjogren’s syndrome, uterine myoma, endometriosis, ovarian cyst, cervical cancer, gastric cancer, thyroid cancer, and malignant lymphoma. Two patients of the M group took oral prednisolone during the follow-up period. The sites for metastasis of M group were brain (3 patients), lung (15 patients), liver (15 patients), and stomach (1 patient). Those of C group were brain (1 patient) and liver (2 patients). The other 29 patients of C group did not have metastasis (Table 3).

**Table 1:** Characteristics, follow-up period, hormonal therapy, and femoral neck-shaft angle of patients

	M group (*n* = 32)	C group (*n* = 32)	*p* value
Age (years)	59.2 ± 11.6	61.9 ± 13.4	0.444
Height (cm)	152.8 ± 6.7	154.4 ± 5.8	0.485
Weight (kg)	54.2 ± 8.8	56.5 ± 9.4	0.452
BMI (kg/m^2^)	23.2 ± 3.6	23.7 ± 3.6	0.677
Follow-up period (months)	56.6 ± 25.7	53.5 ± 27.4	0.528
Luminal type	25 (78.1%)	26 (75.0%)	0.756
HER2-positive type	3 (9.4%)	3 (9.4%)	1.000
Triple-negative type	0	3 (9.4%)	0.119
Unknown	4 (12.5%)	0	0.057
Chemotherapy	20 (62.5%)	9 (28.1%)	0.006
Endocrine therapy	28 (87.5%)	28 (87.5%)	1.000
Neck-shaft angle (right)	140.7 ± 6.4	143.0 ± 5.8	0.056
Neck-shaft angle (left)	140.0 ± 6.7	140.7 ± 6.6	0.453

The values of age, height, weight, BMI, follow-up period, and femoral neck-shaft angle are in mean ± SD

*P* values below 0.05* indicate significant level of difference between the M group and C group, using the Student’s *t* test for parametric data and Mann–Whitney *U* test for nonparametric data

**Table 2:** Types of antiresorptive drugs in the M group

Type of antiresorptive drug	The number of patients
Pamidronic acid, zoledronic acid	1
Zoledronic acid only	12
Zoledronic acid, denosumab	18
Denosumab only	1
Total	32

One patient of the M group underwent pamidronic acid and zoledronic acid therapy, 12 underwent zoledronic acid only, 18 underwent zoledronic acid and denosumab, and 1 underwent denosumab only

**Table 3:** The site for distant metastasis in subjects

	M group (*n* = 32)	C group (*n* = 32)
Brain	3 (9.4%)	1 (3.1%)
Lung	15 (46.9%)	0
Liver	15 (46.9%)	2 (6.3%)
Stomach	1 (3.1%)	0
Bone	32 (100%)	0
No metastasis	0	29 (90.6%)

The numbers of brain, lung, liver, stomach, bone and no metastasis are shown

Of the 64 limbs in 32 patients of the M group, 8 limbs in 6 patients showed beaking at the subtrochanteric area (12.5%). On the other hand, in the C group, no beaking was observed. All 6 patients with beaking underwent bone scintigraphy to exclude bone metastasis. Five of six patients had no uptake of ^99m^Tc and one patient showed uptake in the subtrochanteric area. However, there were no findings of bone sclerosis and osteolysis suggestive of the metastasis on CT scan in this one patient. The period from induction of antiresorptive drugs to the occurrence of beaking was, on an average, 48.4 months (range 22–75 months). After the occurrence of beaking, 5 limbs in 3 patients eventually had a complete fracture with minor trauma (7.8%) (Table 4). There were no patients with rheumatoid arthritis or received steroid treatment. Only 1 limb showed a complete fracture in the distal femoral shaft, and the site of this fracture was out of range of CT evaluation. All of these patients underwent surgery. The period from the presence of beaking to complete fracture was on, an average, 23.0 months (range 17–31 months). Only 1 patient with AFF (16.7%) experienced hypocalcemia during the follow-up period, and 6 (23.1%) patients without AFF experienced hypocalcemia in M group. The mean right femoral neck-shaft angles were 140.7° ± 6.4° in the M group and 143.0° ± 5.8° in the C group, and left were 140.0° ± 6.7° in the M group and 140.7° ± 6.6° in the C group (Table 1). There were no significant differences in right (*p* = 0.056) and left (*p* = 0.453) between the 2 groups. Treatment duration significantly correlated with the presence of beaking (*r* = 0.486, *p* = 0.005); on the other hand, it did not correlate with the occurrence of complete fracture (*r* = 0.285, *p* = 0.114).

**Table 4:** Characteristics of patients with AFF

Patient	Age (years)	Side	Metastasis (excluding bone)	Drugs	Duration (months)	Response to therapy	Beaking (months)	Fracture (months)
A	50	Right	Liver	Z	74	SD	42	–
B	53	Right	Liver	Z, D	78	PD	62	–
B	53	Left	Liver	Z, D	78	PD	64	–
C	58	Right	–	P, Z	89	SD	41	68
C	58	Left	–	P, Z	89	SD	46	77
D	64	Right	–	Z	100	PD	35	52
E	68	Right	–	Z, D	101	PD	75	–
F	73	Right	–	Z, D	54	SD	22	39
F	73	Left	–	Z, D	54	SD	UNK	45

The period to beaking and complete fracture from induction of antiresorptive drug therapy is shown. Eight limbs in 6 patients showed beaking at the subtrochanteric area (12.5%). The period from induction of antiresorptive drugs to the occurrence of beaking was, on average, 48.4 months. After the occurrence of beaking, 5 limbs in 3 patients eventually had a complete fracture from minor trauma (7.8%). The period from the presence of beaking to a complete fracture was, on an average, 23.0 months. Patient F underwent AFF on the supracondylar of the left side, and this area was out of CT evaluation. Therefore, the occurrence time of beaking in the left femur was unknown. Bone metastasis’s response to therapy was measured as progressive disease (enlargement of metastatic lesion on CT scan) or stable disease (no enlargement of metastatic lesion on CT scan)

*D* denosumab, *P *pamidronic acid, *Z* zoledronic acid, *PD* progressive disease, *SD* stable disease, *UNK* unknown

## Discussion

This study showed that the occurrence rate of AFF in patients with breast cancer receiving antiresorptive therapy for bone metastasis was higher than that of those who did not receive antiresorptive drugs. Of those who received antiresorptive therapy, 8 limbs in 6 patients (12.5%) showed beaking in the subtrochanteric area and 5 limbs in 3 patients (7.8%) had a complete fracture. There are several studies on the frequency of AFF in patients using bisphosphonate for osteoporosis. Schilcher J et al. investigated 12,777 Swedish women using bisphosphonate for osteoporosis and reported 59 patients with AFF [11]. Black DM et al. reviewed 3 clinical trials and reported 12 of 14,195 patients with AFF [12]. As mentioned above, most previous reports about AFF were intended for the patients receiving lower doses of bisphosphonate typically used to treat osteoporosis. Two case series reported the frequency in patients with cancer. Puhaindran et al. investigated 327 patients with skeletal malignant involvement who received a minimum of 24 doses of intravenous bisphosphonates and reported 4 cases of AFF [13]. Chang et al. who investigated 62 patients with breast cancer or multiple myeloma with a femur fracture and prior intravenous bisphosphonate treatment for bone malignancy, reported 6 cases of AFF [14]. The frequency of AFF may have been higher (12.5%) in our study because only breast cancer patients were subjects of investigation and all patients underwent CT scans to diagnose AFF.

The beaking appeared, on an average, at 48.4 months after antiresorptive therapy induction in patients with breast cancer and bone metastasis. On the other hand, no beaking was observed in those who did not undergo antiresorptive drugs in this study. There have been several reports on the period of fracture after bisphosphonate induction. Park-Wyllie et al. explored the association between bisphosphonate and fractures in a cohort of elderly women using bisphosphonate. They mentioned that among these elderly women, treatment with a bisphosphonate for more than 5 years was associated with an increased risk of subtrochanteric or femoral shaft fractures [15]. Also, there have been a few case reports for denosumab [16]. The mechanism of AFF and its association with antiresorptive drugs remains unknown. One hypothesis is that the chronic suppression of bone turnover by bisphosphonates may lead to an accumulation of microdamage in the bone, weakening and eventually leading to a fracture [3]. Therefore, there is an interval of several years from induction of antiresorptive therapy to the occurrence of AFF.

In this study, there was no significant association between the femoral neck-shaft angle and the occurrence of AFF. The cause of AFF is unknown and likely multifactorial. The biomechanical factor is one of the reasons for AFF. Hagen et al. mentioned that patients who presented with AFF had more varus proximal femoral geometry than those who did not sustain a fracture [17]. Oh et al. reported that of the 12 cases of low-energy femoral shaft fractures associated with bowing deformity, 6 cases were not treated with bisphosphonate at all [18]. They said that stress fractures associated with a femoral shaft bowing deformity should be recognized as another cause of AFF. In this study, there was no difference in the femoral neck-shaft angle between patients with AFF and without AFF in the M group. However, it is possible that our measurements overestimated or underestimated the actual varus in our patients because of limb rotation.

**Fig. 2:**
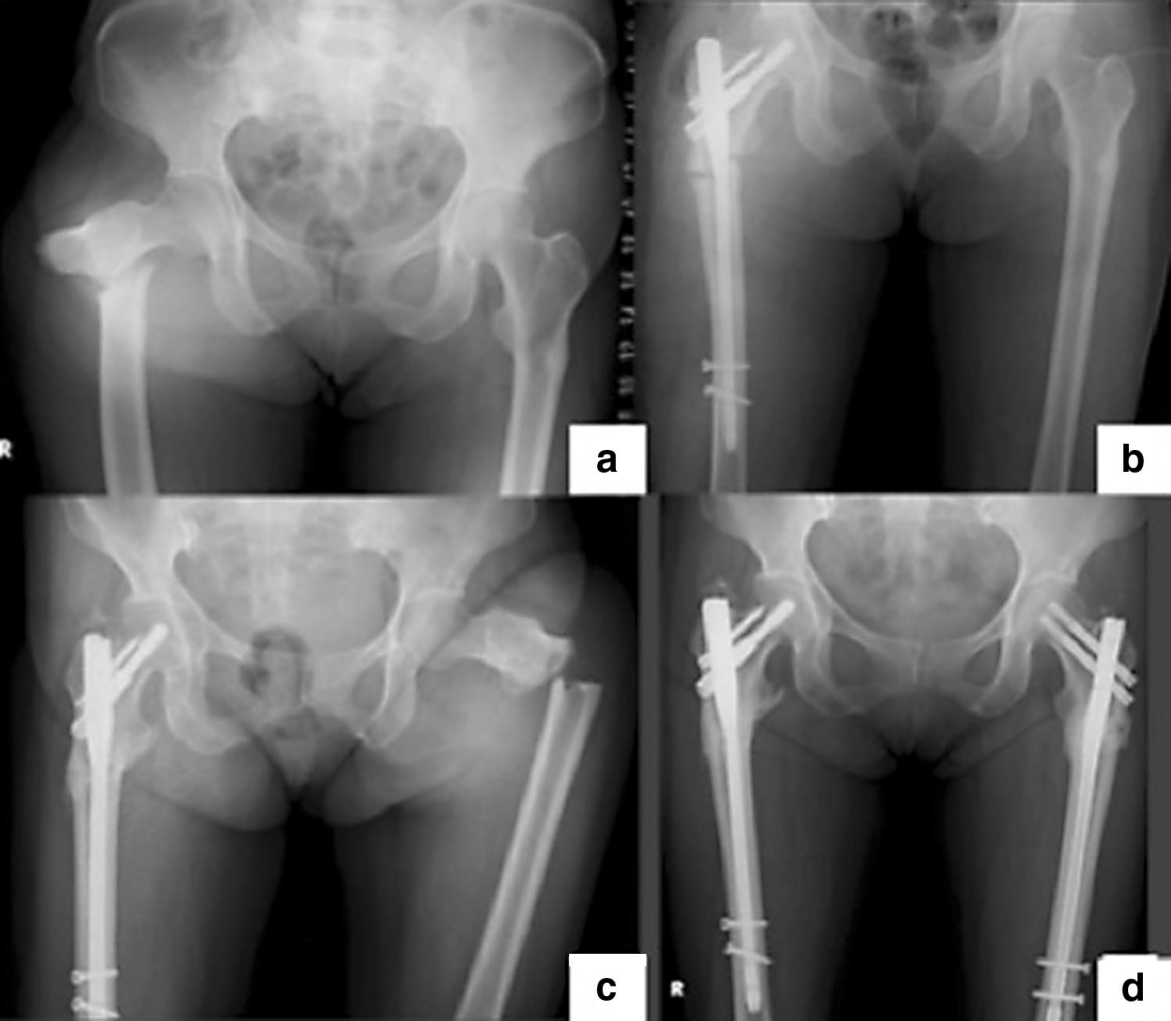
Complete fracture of the bilateral femur by AFF. **a** Complete fracture of the right femur and beaking on the contralateral side. **b** Post open reduction and internal fixation of right femur. **c** Complete fracture of the left femur by AFF. **d** Post open reduction and internal fixation of the left femur

In regard to the complete fracture after beaking occurrence, this study showed that 3 of 5 patients with beaking definitely resulted in complete fracture (Fig. 2). There have been few reports about the prevalence rate of complete fracture after beaking occurrence due to AFF. However, in general, all incomplete AFF may progress to a complete fracture. Ha et al. mentioned that femoral insufficiency fractures after prolonged bisphosphonate therapy seldom healed spontaneously and most patients underwent surgery for fracture displacement or persistent pain [19]. Shane et al. reported that if there is no symptomatic and radiographic improvement after 2–3 months of conservative therapy, preventive nail fixation should be strongly considered as these patients may progress to a complete fracture [1].

Although the number of reports about the surgical treatment of AFF is increasing, it is unclear if a preventive surgery is needed or useful in incomplete fractures [20]. Several reports recommended conservative therapy that involves replacing bisphosphonate with teriparatide and limiting weight-bearing through the use of crutches or a walker. However, these conservative therapies have a significant negative impact on quality of life in these patients, and in a cancer patient, teriparatide is a contraindication. If a complete fracture occurs, the patient may require surgery and rehabilitation [21, 22]; therefore, the prevention of complete fracture is important. Routine X-rays should be undertaken to investigate any signs and symptoms associated with AFF. Preventive surgery may be effective to avoid complete fracture. Chang et al. retrospectively investigated 20 incomplete, non-displaced AFF with intramedullary nailing. They concluded that preventive fixation of AFF is recommended [14]. In this study, complete fracture after the appearance of beaking was seen in 5 of 9 limbs. Only 1 patient (1 limb) underwent preventive intramedullary nailing before the occurrence of complete fracture because transverse cortical fracture line inside beaking was detected, which was a possible precursor lesion of complete fracture. Of the complete fractures, 2 patients (4 limbs) received intramedullary nailing and 1 patient (1 limb) received hemiarthroplasty. Bony union was observed 6–12 months after the operation. It is possible that preventive intramedullary nailing in patients with beaking is necessary.

This study had several limitations. First, as bone biopsy was not performed, histopathological examination was not studied. Second, bone mineral density measurements were not taken for the patients, which would have helped us to determine whether these patients had decreased bone density, which is a major risk factor for fragility fracture. Third, the range of CT was from the neck level to proximal thigh level; thus, not all of the femoral shaft was investigated.

In spite of above limitations, this study showed that the frequency of AFF in patients with breast cancer receiving bisphosphonate and/or denosumab for bone metastasis was higher than those who did not undergo antiresorptive drugs. Also, the rate of complete fracture after the appearance of beaking was high. Therefore, more attention should be paid to the occurrence of AFF in these patients than osteoporotic patients, and it is possible that preventive surgery for AFF before a complete fracture is necessary.
